# The *ter* Mutation in the Rat *Dnd1* Gene Initiates Gonadal Teratomas and Infertility in Both Genders

**DOI:** 10.1371/journal.pone.0038001

**Published:** 2012-05-24

**Authors:** Emily Northrup, Nils-Holger Zschemisch, Regina Eisenblätter, Silke Glage, Dirk Wedekind, Edwin Cuppen, Martina Dorsch, Hans-Jürgen Hedrich

**Affiliations:** 1 Institute of Laboratory Animal Science, Hannover Medical School, Hannover, Germany; 2 Hubrecht Institute, Royal Netherlands Academy of Arts and Sciences and University Medical Center Utrecht, Utrecht, The Netherlands; University of Tampere, Finland

## Abstract

A spontaneous mutation leading to the formation of congenital ovarian and testicular tumors was detected in the WKY/Ztm rat strain. The histological evaluation revealed derivatives from all three germ layers, thereby identifying these tumors as teratomas. Teratocarcinogenesis was accompanied by infertility and the underlying mutation was termed *ter*. Linkage analysis of 58 (WKY-*ter*×SPRD-*Cu3*) F2 rats associated the *ter* mutation with RNO18 (LOD = 3.25). Sequencing of candidate genes detected a point mutation in exon 4 of the *dead-end homolog 1 gene (Dnd1*), which introduces a premature stop codon assumed to cause a truncation of the Dnd1 protein. Genotyping of the recessive *ter* mutation revealed a complete penetrance of teratocarcinogenesis and infertility in homozygous *ter* rats of both genders. Morphologically non-tumorous testes of homozygous *ter* males were reduced in both size and weight. This testicular malformation was linked to a lack of spermatogenesis using immunohistochemical and histological staining. Our WKY-*Dnd1^ter^*/Ztm rat is a novel animal model to investigate gonadal teratocarcinogenesis and the molecular mechanisms involved in germ cell development of both genders.

## Introduction

Tumors classified as germ cell tumors (GCTs) are derived from cells belonging to the germline and are a heterogeneous group of neoplasms with varying histopathological and clinical manifestations [1]. Human germ cell tumors (GCT) can be observed in all age groups, ranging from infancy to adulthood, and are frequently diagnosed at a young age [2], [3]. To date, the molecular principles underlying ovarian germ cell tumor (OGCT) development remain poorly understood [4], [5]. However, in the testes mutations in *TP53, KIT, KRAS/NRAS*, and *BRAF* genes can contribute to the neoplastic transformation of germ cell precursors [6]. Furthermore, recent genome-wide association studies linked six gene loci to the development of testicular germ cell tumors (TGCTs): *KITLG, SPRY4* and *BAK1* are involved in the KIT pathway, *TERT-CLPTM1L* and *ATF7IP* in telomerase regulation, and *DMRT1* in sex determination [7], [8], [9]. A review of transcriptome studies on TGCT identified 93 recurring genes involved in TGCT, including relevant cancer genes (e.g. KRAS, MYCN) and pluripotency-associated genes such as the embryonic transcription factors *NANOG* and *POU5F1* [10]. Various male reproductive disorders, including infertility and TGCTs, are thought to result from similar disruptions during fetal development and have been grouped together in the so-called testicular dysgenesis syndrome (TDS) [11], [12].

Teratocarcinomas are malignant GCTs consisting of derivatives from all three germ layers and undifferentiated embryonic carcinoma cells (ECCs). The pluripotent ECCs are the stem cells of GCTs that are capable of differentiating into the various tumor tissues [13] and originate from a neoplastic transformation of germ cells during embryonic development [14], [15]. Primordial germ cells (PGCs) are the embryonic precursors of the adult gamete and they migrate through various embryonic tissues before reaching the genital ridge, after which they are classified as gonocytes or late PGCs. In general, teratomas can be grouped into prepubertal type I GCTs and the more common postpubertal type II GCTs. Type I GCTs have a biparental partially erased pattern of genomic imprinting suggesting that the precursor cells are earlier PGCs or gonocytes that are directly transformed into ECCs. Genomic imprinting is erased in the aneuploid type II GCTs and probably involves late PGCs/gonocytes that are transformed into precursor lesions (CIS/ITGCN) before developing into ECCs [16], [17], [18].

Spontaneous formation of testicular teratomas was described in the 129/Sv mouse strain in the early 1950ies [19] and these tumors can be accompanied by metastases [20]. TGCT development was detected in 1% of male 129/Sv mice, while an additional mutation in a modifier gene referred to as *Ter* increased the incidence of TGCT to 17% in *Ter*/+ and to 94% in *Ter/Ter* males. Teratocarcinogenesis in the male 129/Sv-*Ter* mice was associated with infertility in male and decreased fertility in *Ter/Ter* female mice [21], which has been attributed to the embryonic loss of PGCs [22], [23]. The *Ter* mutation was identified on mouse chromosome 18 as a C to T substitution in the *Dnd1* gene introducing a premature stop codon and leading to functional inactivation [24], [25]. The dead end gene is thought to play an evolutionary conserved role in germ cell development, and is also required for PGC migration and survival in the anamniotic species *Danio rerio* and *Xenopus laevis* [26], [27], [28].

Dnd1 is an RNA-binding protein (RBP) that competes with microRNAs for the binding of target gene mRNAs, as shown in human and mouse cells. It inhibits the miRNA-mediated repression of the tumor suppressor genes *p27* and *Lats2*, the germ cell specific genes *Nanos1* and *Tdrd7* as well as *Connexin43 (Gja-1)* [29], [30]. Moreover, Dnd1 is postulated to form complexes with APOBEC3, a multifunctional protein also involved in the inhibition of miRNA-mediated mRNA repression [31]. Loss of *Dnd1* in male germ cells induces a lower expression of male differentiation genes, the upregulation of meiotic markers, the preservation of pluripotency genes and the inability of mutant germ cells to enter mitotic arrest at G0 [32]. The Dnd1 protein associated with transcripts encoding pluripotency factors, cell cycle regulators and apoptotic factors in embryonic stem cells [33]. Apoptosis is at least in part responsible for the germ cell loss, as additional inactivation of the pro-apoptotic *Bax* gene in *Dnd1^Ter/Ter^* mice rescued up to 50% of the PGCs in both genders [34]. The key to understanding GCTs and infertility lies in discerning factors and genetics regulating germ cell differentiation to mature gametes, de-differentiation to pluripotent cells and apoptosis. *Dnd1* assumes a vital role in these processes in the mouse.

In 2004 our group reported a spontaneous recessive mutation in WKY/Ztm rats referred to as *ter* that initiated congenital teratomas in the rat testes and ovaries [35]. We have shown that the homozygous *ter* mutation facilitates the transformation of germ cells from embryonic day 14.5 post coitum (pc) into pluripotent cells in culture, whereas the cultivation of germ cells from embryonic day 10.5 pc is not influenced [36].

Here we are able to report the identification of *ter* as a point mutation in the rat *Dnd1* gene. This mutation inserts a premature stop codon leading to the loss of the c-terminus of the Dnd1 protein. Unlike the mouse, *Dnd1* is found to be essential for survival and inhibition of the neoplastic transformation of germ cells in every rat of either gender from the WKY-*Dnd1^ter^*/Ztm strain.

## Materials and Methods

### Animals

All animals were bred and maintained at the Institute for Laboratory Animal Science, Hannover Medical School (MHH), Germany (subline code: Ztm: http://www.mh-hannover.de/einrichtungen/tierlabor). The MHH has the PHS approved Animal Welfare assurance number A5919-01. The experiments were in accordance with the German Animal Welfare Legislation (Tierschutzgesetz 2006) and reported to the Lower Saxony State Office for Consumer Protection and Food Safety (LAVES).

### Husbandry

The WKY/*Dnd1^ter^/*Ztm (further abbreviated as WKY-*ter*) colony is maintained as a segregating inbred strain by mating littermates or parents known to carry the mutation. Microbiological status was monitored according to FELASA recommendations [37] and the WKY/Ztm rats were positive for parvovirus and apathogenic protozoa. Rats were kept in groups of three animals under a 14∶10 light-dark cycle and 55±5% humidity. They received an autoclaved commercial pelleted diet (Altromin 1314, Altromin; Lage, Germany) (protein 22%, fat 5%, raw fiber 4.5%, ash 7%, utilizing energy 3.1 kcal/g) and water *ad libitum*. The commercial softwood granulate bedding was sterilized (Lignocel, Altromin).

### Linkage analysis

We created an F1 generation by mating heterozygous male WKY-*ter* rats with female SPRD-*Cu3* rats to identify teratocarcinoma susceptibility loci. SPRD-*Cu3* was chosen, because it is easily distinguished from the WKY-*ter* strain by microsatellites. The F1 generation was used to generate a [WKY-*ter*×SPRD-*Cu3*] F2 generation (n = 58). All offspring were monitored daily for tumors by inspection and palpation of the scrotum and the abdominal cavity. The incidence of teratomas in the F2 population was 15%.

### DNA samples

Genomic DNA was extracted from tissues using the NucleoSpin™ Tissue kit (Macherey-Nagel, Dueren, Germany) according to the manufacturer's instructions.

### Microsatellite analyses

All oligonucleotides used in this study were synthesized by MWG Biotech AG (Ebersberg, Germany). The microsatellite markers were selected using rat genome maps published by the Whitehead Institute, the Rat Genome Database and Ratmap.

All primers were tested against a panel of the progenitor strains and the F1 generation to determine the polymorphic nature of the microsatellite markers. Approximately 52 microsatellite markers proved to be polymorphic between WKY-*ter* and SPRD-*Cu3* and were therefore used in a genome wide screen of the F2 progeny. A complete list of all used microsatellites can be requested from the authors. The PCR reaction (4 min at 94°C; 35 cycles: 15 s at 94°C, 1 min at 55°C, 2 min at 72°C; 7 min at 72°C) was performed in a PTC-200 thermocycler (Biozym, Hess. Oldendorf, Germany) with 100 ng DNA template per well in a 96-well plate (MultiRigid Ultra Plates™, Roth, Karlsruhe, Germany). PCR products were analyzed by electrophoresis in 3% Nusieve™ agarose gels (Biozym) and stained using Gelstar™ (Cambrex, Apen, Germany).

### SNP analysis and sequencing

A panel of 64 SNP markers, which are polymorphic between WKY and SPRD-*Cu3,* was selected. Resequencing of SNPs and all four *Dnd1* exons was managed using LIMSTILL, LIMS for Induced Mutations by Sequencing and TILLing (Victor Guryev, E.C., unpublished). This web-based publicly accessible information system (http://limstill.niob.knaw.nl) was used to generate the project and visualize the *Dnd1* gene structure based on the Ensembl file ENSRNOG00000016894. The primer design application within LIMSTILL is Primer3-based, and parameters are set to design primers with an optimal melting temperature of 58°C.

The PCR for *Dnd1* was performed using a touchdown thermocycling program (92°C for 60 s; 12 cycles: 92°C for 20 s, 65°C for 20 s with a decrement of 0.4°C per cycle, 72°C for 30 s; 20 cycles: 92°C for 20 s, 58°C for 20 s and 72°C for 30 s and 72°C for 180 s; GeneAmp9700, Applied Biosystems; Foster City, CA). PCR reaction mixes contained 5 µl genomic DNA, 0.2 µM of forward/reverse primer, 200 µM of each dNTP, 25 mM Tricine, 7.0% Glycerol (w/v), 1.6% DMSO (w/v), 2 mM MgCl_2_, 85 mM Ammonium acetate pH 8.7 and 0.2 U Taq Polymerase ad 10 µl. PCR products were diluted with 20 µl H_2_O, and 1 µl was used as template for sequencing with 0.25 µl BigDYE (v1.1; Applied Biosystems), 3.75 µl 2.5× dilution buffer (Applied Biosystems) and 0.4 µM gene specific primer in a total volume of 10 µl using cycling conditions as recommended by the manufacturer. Sequencing products were purified by ethanol precipitation in the presence of 40 mM sodium-acetate and analyzed on a 96-capillary 3730XL DNA analyzer (Applied Biosystems). Sequences were analyzed for polymorphisms using PolyPhred software [38]. Primers for PCR amplification and sequencing were designed using the Ensembl genome database (http://www.ensembl.org) and a customized interface to Primer3.

### Genotyping

To identify the *ter* allele by PCR, primers terFor2 5′-GTCTGGTCTTAAGTGCTTGG-′3 and terRev2 5′-TCACTGCTTCACCACAGAAC-3′ amplified a 560 bp sequence in a total volume of 25 µl containing 12.5 µl HotStarTaq Master Mix Kit (Qiagen, Hilden, Germany), 10 pmol of each primer and 1 µl template DNA (15 min at 95°C; 35 cycles: 95°C for 30 s, 54°C for 30 s, 72°C for 30 s; 72°C for 1 min). The PCR products were digested by adding 0.5 µl *Kpn*I-HF, 3 µl NEB4 buffer and 0.3 µl BSA (100×) (NEB, Ipswich, MA). Cleavage fragments from PCR products of wild type *Dnd1* (180 bp and 380 bp) and unchanged 560 bp *ter* allele products were separated by electrophoresis in a 1.5% agarose gel in 1× TBE buffer.

### Tumors and tissues

GCTs were harvested from 363 male and 520 female *Dnd1*-deficient rats at the pre-final stage of cancer, which were characterized by a significant increase of body weight accompanied by abdominal swelling, inactivity, reduced food intake or piloerection/rough coat. Tumors were excised, examined macroscopically, weighed and prepared for histology. Organs were evaluated for metastatic spread.

In addition, ten males and ten females from the WKY/Ztm strain as well as from the WKY-*Dnd1^ter^*/Ztm strain with +/+, *ter*/+ and *ter*/*ter* genotype were sacrificed at 3, 6 and 9 weeks of age by cervical dislocation after CO_2_ anesthesia. Testes and ovaries were evaluated macroscopically, weighed, and fixed for histology with some tissue being saved for RNA isolation. The 1-, 5- and 10-day-old animals were sacrificed by decapitation, and the gonads prepared for histological examination using microsurgical instruments.

### Histology and Immunohistochemistry

Tissues were fixed in 4% formaldehyde solution or in Bouin's solution (Sigma-Aldrich, St.Louis, MO), paraffin-embedded, sectioned at 3 µm, and transferred to SuperFrost slides (Menzel, Braunschweig, Germany). Staining with hematoxylin-eosin followed standard procedures.

For immunohistochemical staining against c-kit, slides were heated in 10 mM citric buffer (pH 6) for 5 min at 125°C in a pressure cooker for antigen retrieval. Samples were stained with a 1∶100 dilution (stock 200 µg IgG/ml PBS) of an anti-c-kit antibody (sc-168; Santa Cruz Biotechnology Inc, Santa Cruz, CA) for 1 hour. For the anti-vasa staining, slides were heated to 96°C for 10 min in 10 mM citric buffer (pH 6) in a water bath and incubated with a 1∶200 dilution of an anti-DDX4/MVH antibody (1 mg/ml, ab13840; Abcam, Cambridge, UK). The secondary antibody reaction and HRP staining were performed using the Zytochem Plus HRP Polymer System (Zytomed Systems, Berlin, Germany) according to the manufacturer's instructions.

### Statistical analyses

Linkage analyses were performed with the JoinMap V 2.0 program (Agricultural Research Department, Wageningen, Netherlands). The LOD scores of the teratocarcinoma susceptibility region were calculated using the R/qtl program provided by Dr. K. Browman (Department of Biostatistics, Johns Hopkins University, Baltimore, MD) [39], [40]. E/M algorithms estimated susceptibility regions in a binary model using the teratocarcinoma of the animals as a trait. A permutation test was performed based on our current genotypic and phenotypic data to calculate an individual threshold value for significance (LOD score >2.3) independent from the theoretical model of Lander and Kruglyak [41], [42]. Tumor data analysis and Kaplan-Meyer survival analysis were performed and statistically verified using One-way Anova and Bonferroni Multiple Comparison Test by the PRISM 5 for Mac OSX Software (GraphPad Software Inc., La Jolla, CA).

### *Dnd1* expression analysis

RNA was prepared from animal tissues with the Rneasy Mini Kit (Qiagen) following the suppliers recommendations. Quantitect Reverse Transcription Kit (Qiagen), which eliminates contaminating genomic DNA, was used for cDNA synthesis from 1 µg RNA. *Dnd1* expression was analyzed in quantitative Real Time PCR assay (qRT-PCR) with the primers qDnd1for 5′-GCTTGAACCGACGTGCT-′3 and qDnd1rev 5′-TGCTAAACTTGAGCAGTGCAATTTG-′3, which avoid the processed *Dnd1* pseudogenes located on chromosomes 4 and 20 as well as the X chromosome (GeneID: 688733). The PCR products of *Dnd1* were amplified in the StepOne Real Time PCR System (Applied Biosystems) using the TaqMan® Gene Expression Mastermix (Applied Biosystems). Detection of the *Dnd1* PCR products were performed with the MGB Taq Man probe 3′-CAGGAGACATTGCTGC-′5 labeled with FAM and a non-fluorescent quencher (Applied Biosystems). *GAPDH* expression was used as endogenous control with the Rat GAPD (*GAPDH*) Endogenous Control (VIC®/MGB Probe, Primer Limited; Applied Biosystems).

*Dnd1* transcription rate was measured in cDNAs synthesized from RNA of the gonads at 3, 6 and 9 weeks of age. A rat liver cDNA served as reference sample.

### Cloning and *in vitro* protein expression

*Dnd1^ter^* and *Dnd1^wt^* cDNAs were amplified using the OneTaq® Hot Start 2× Master Mix with Standard Buffer (NEB) with primers Dnd1for1 5′-ATGCAGTCCAAACGGGAGTG-′3 and terRev2 5′-TCACTGCTTCACCACAGAAC-3′ at 94°C for 30 sec; 35 cycles: 94°C for 30 sec, 60°C for 20 sec, 68°C for 1 min; 68°C for 5 min. The cDNAs were directly cloned into Vivid Colors™ pcDNA™6.2/N-EmGFP-GW/TOPO® Mammalian Expression Vector (Life Technologies, Darmstadt, Germany) for expression of GFP-Dnd1^wt^ or GFP-Dnd1*^ter^* fusion proteins, which were verified by sequencing. The vectors containing wild type or *ter Dnd1* cDNA were transfected into Huh7 human hepatoma cells with the X-treme GENE HP DNA Transfection Reagent (Roche, Mannheim, Germany) following the manufacturer's recommendations. Cells were harvested 24 hours after transfection and proteins were isolated using NP40 buffer.

Furthermore, the GFP-Dnd1 cDNA was amplified using the primers EmGFPfor: 5′-AGACGTTGTGGCTGTTGTAG-′3 and terRev2: 5′-TCACTGCTTCACCACAGAAC-3′ with OneTaq® Hot Start 2× Master Mix with Standard Buffer (NEB) at 94°C for 30 sec; 35 cycles: 94°C for 30 sec, 53°C for 20 sec, 68°C for 1 min; 68°C for 5 min; and digested with *Kpn*I.

### Immunoblotting

GFP-Dnd1 fusion protein was detected in Western Blot Analysis with a 1∶1000 dilution of a rabbit polyclonal anti-GFP antibody (#2555; Cell Signaling Technology, Danvers, MA) and a 1∶250 dilution of the Rabbit polyclonal anti-rat Dnd1 antibody detecting the N-terminal RLVQVNGQRKYGGPP epitope (aa 31–45) (Eurogentec, Seraing, Belgium) with a 1∶5000 dilution of the donkey anti-rabbit IgG-HRP secondary antibody (ab16248; Abcam). As endogenous control rat GAPDH protein was detected with a 1∶500 dilution of the mouse monoclonal anti-GAPDH antibody (MCA4740; AbD Serotec, Duesseldorf, Germany) and a 1∶5000 dilution of the F(ab′)2 rabbit anti-mouse IgG: HRP secondary antibody (AbD Serotec).

## Results

We traced the *ter* mutation in the WKY/Ztm rat strain to a point mutation in the *Dnd1* gene and observed teratocarcinomas and infertility in all homozygous rats of both genders.

### Teratocarcinomas in the WKY strain

**Figure 1:**
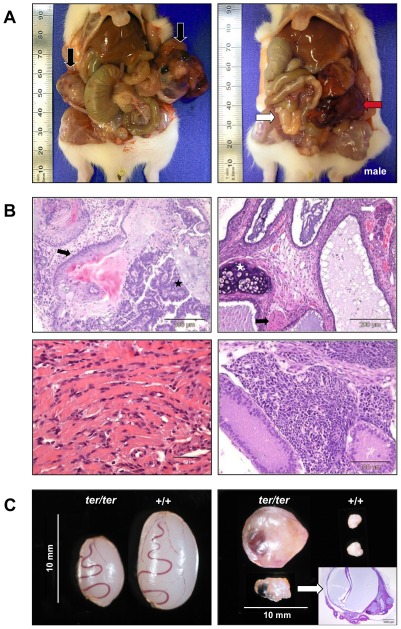
Teratomas and gonads in *ter/ter* rats. (**A**) Left: Bilateral GCTs in a 6 week old female rat (black arrows: OGCTs). Right: Unilateral GCT in male rat (white arrow: non-neoplastic testes; red arrow: left TGCT). (**B**) HE staining of teratomas. Top left: ectodermal tissues in the ovarian teratoma of a 5-week-old female; neural tube formation (black star) and squamous epithelium (black arrow). Top right: mesodermal and endodermal tissues in the ovarian teratoma of a 9-week-old female; cartilage (white star), glandular structures (white arrow) and skeletal muscle (black arrow). Bottom left: heart muscle-like tissue from a contractile ovarian teratoma. Bottom right: immature neuronal tissue from a testicular teratoma of a 6-week-old male. (**C**) Rat gonads from 3-week-old siblings. Left: non-tumorous *ter/ter* testes are significantly smaller than wild type. Right: physiological wild type ovaries compared to an ovarian teratoma and an abnormal, cystic ovary (confirmed by histological section and HE staining).

We established a coisogenic segregating inbred WKY strain that carries a mutation referred to as *ter* leading to germ cell tumors (GCT) in both testes and ovaries (Fig. 1A). Histological examination of these tumors revealed tissues originating from all three germ layers, thereby characterizing the GCTs as teratomas. Aside from mesodermal tissues such as cartilage, skeletal and cardiac muscle, we also identified glandular structures arising from the endodermal germ layer. Furthermore, immature neuronal tissue, neural tube-like formations and squamous epithelia originating from the ectoderm were present in teratomas of both ovaries and testes (Fig. 1B).

In most cases tumor development was accompanied by abdominal adhesions, hemorrhagic ascites and/or tumor cachexia. Moreover, the early stage of tumorigenesis seemed to be associated with cystic alterations of the ovary (Fig. 1C, left).

### Identification of the *ter* mutation

**Figure 2:**
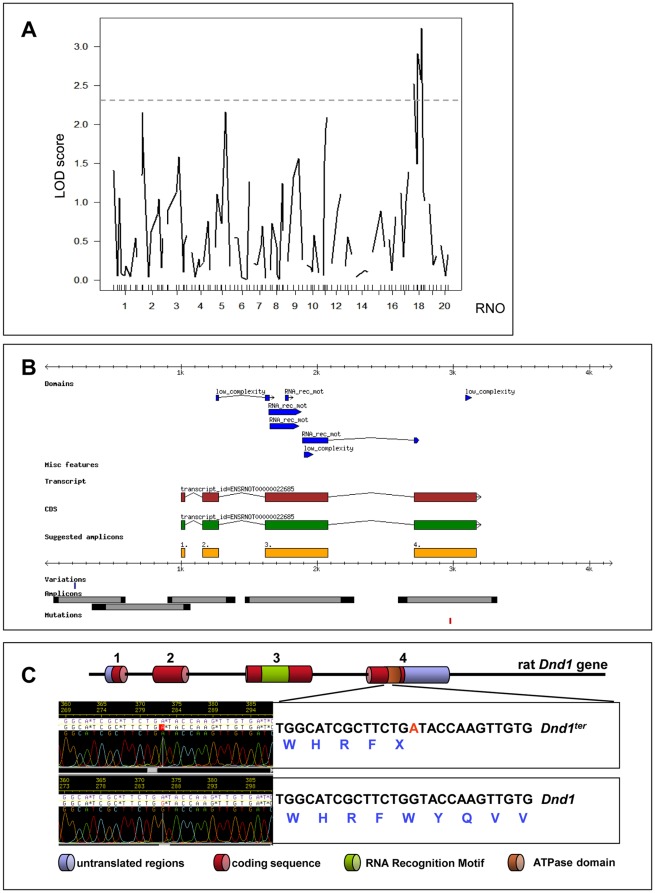
Identification of the *ter* mutation in the rat *Dnd1* gene. (**A**) Genome-wide mapping of teratoma susceptibility loci of the WKY-*ter* rat. 58 (WKY-*ter*×SPRD-*Cu3*) F2 animals were typed using genomic DNA and the complete panel of polymorphic microsatellite markers. The LOD scores of the teratoma susceptibility region were calculated using the R/qtl program. A permutation test assumed a LOD score >2.3 to be associated with teratoma development. (**B**) LIMSTILL was used to generate the *Dnd1* project, manage the resequencing, and visualize the gene structure based on Ensembl file ENSRNOG00000016894. (**C**) The 2625 bp coding sequence of the *Dnd1* gene is separated in 4 exons. The RNA Recognition Motif (RRM) essential for nucleic acid binding of *Dnd1* was located in exon 3. The *ter* point mutation was identified as G to A substitution in exon 4 at position 1975 introducing a premature stop codon.

Linkage analysis of 58 (WKY-*ter*×SPRD-*Cu3*) F2 rats using 52 polymorphic microsatellite markers showed an association of RNO18 (*D18Rat61,* LOD = 3.25) with tumor development (Fig. 2A). Additionally, a panel of 64 SNP markers, which are polymorphic between WKY and SPRD-*Cu3,* was selected to verify these findings. The genomic loci were amplified by PCR from the 58 F2 animals of the WKY-*ter*×SPRD-*Cu3* cross and genotyped by dideoxy re-sequencing. Strong linkage to a single locus in the middle of chromosome 18 was observed (data not shown).

Based on synteny with the mouse *Ter* mutation within MMU18qB2, the homologous rat *Dnd1* gene in RNO18p11 was re-sequenced (Fig. 2B). A G to A mutation was identified in exon 4 at position 1975, which introduces a premature stop codon thought to result in a 62 amino acid truncation at the c-terminus of the Dnd1 protein (Fig. 2C).

**Figure 3:**
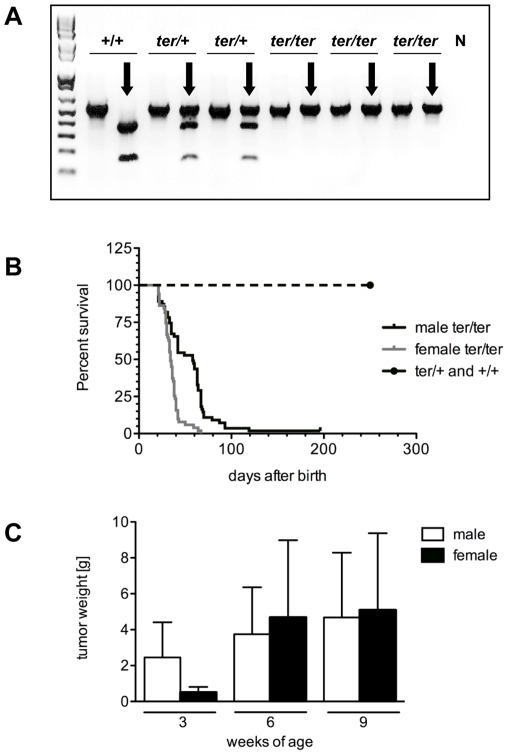
Genotyping, survival and tumor progression. (**A**) Genotyping. The *ter* mutation disrupts a *Kpn*I restriction site used for genotyping of the *ter* allele performing PCR amplification and restriction digest. *Kpn*I digest (black arrows) cleaved the PCR product of 560 bp into 380 bp and 180 bp fragments, N negative control. (**B**) Kaplan-Meyer survival analysis of male and female WKY-*Dnd1^ter^*/Ztm rats carrying heterozygous and homozygous *ter* alleles compared to wild type animals. (**C**) Sizes of TGCTs and OGCTs after 3, 6 and 9 weeks of age in homozygous WKY-*Dnd1^ter^*/Ztm rats.

The *ter* point mutation also disrupts the recognition site of the restriction endonuclease *Kpn*I. A 560 bp PCR amplification product of wild type *Dnd1* was cleaved by *Kpn*I into a 380 bp and a 180 bp fragment, while no cleavage was detected in homozygous *ter/ter* animals, which confirms the absence of the *Kpn*I restriction site (Fig. 3A). Using this genotyping approach, we were able to distinguish between rats carrying only one *ter* allele (*ter/+*), and animals carrying two *ter*-alleles (*ter/ter*), and wild type (*+/+*) animals. The *ter/+* animals showed normal fertility in both genders and were used to establish the coisogenic WKY-*Dnd1^ter^* strain. Litters from breeding heterozygous rats were of the same size as litters from wild type matings of WKY-*Dnd1^ter^* or WKY. Offspring from *ter/+* parents exhibited normal Mendelian ratios with 22.8%+/+, 53.7% *ter*/+ and 24.5% *ter/ter* genotypes.

### Age-dependent GCT development

Female *ter/ter* rats survived 16 to 135 days after birth with a median survival of 35 days, and only 20% of *ter/ter* females exceeding these 35 days of age (Fig. 3B). Male *ter/ter* had to be euthanized starting with day 13 after birth. Of the male *ter/ter* rats 80% had to be sacrificed by day 60 due to tumorigenesis. However, 20% of the *ter/ter* males exhibited a delayed TGCT formation and survived up to 288 days, before exhibiting visual teratomas. Altogether, the median survival was 45 days in males. In contrast to the 100% tumor incidence in *ter/ter* rats, tumorigenesis was entirely absent in the gonads of heterozygous animals. Furthermore, there was no spontaneous teratocarcinogenesis detectable in wild type WKY-*Dnd1^ter^* or WKY rats (Fig. 3B).

Tumor size and localization were examined in male and female rats at 3, 6 and 9 weeks of age to determine tumor progression. The mean TGCT weight increases in *ter/ter* male rats from 2.5 g (SD±2.32) at 3 weeks to 3.8 g (SD±2.96) and 4.7 g (SD±3.76) at 6 and 9 weeks (Fig. 3C). At 3 and 6 weeks of age, 40% of the homozygous *ter* rats had unilateral TGCTs, while both testes were affected in 20%, and in 40% both testes were macroscopically degenerated without signs of tumor formation. At 9 weeks of age bilateral teratomas increased to 68%, while 18% had unilateral tumors and 14% degenerated testes.

At 3 weeks of age the OGCTs in female *ter/ter* rats were smaller (0.54 g±0.38) than the TGCTs. Rapid growth of the OGCTs was detected from 3 to 6 and 9 weeks of age up to mean weights of 4.68 g (SD±4.58) and 5.11 g (SD±4.86), with large variations in tumor size (Fig. 3C). Only 4 females survived up to 9 weeks of age and 3 of these females had bilateral OGCTs. Only 1 of the surviving 4 females at 9 weeks of age exhibited unilateral teratocarcinogenesis, however, the non-tumorous ovary was cystic (Fig. 1C, right). This was the only case of a female *ter/ter* rat developing a teratoma in one ovary and not in both. Ovary degeneration in *ter/ter* females without tumor formation was never found due to the prepubertal onset of tumor development. The rapid tumor progression was correlated to a fast drop of survival between 3 and 6 weeks of age compared to male *ter/ter* rats (Fig. 3C).

**Figure 4:**
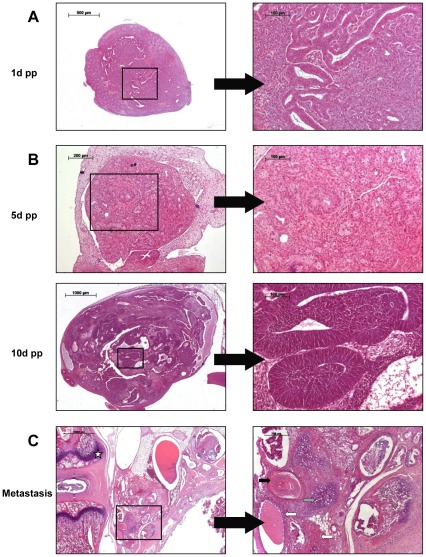
HE staining of early tumorigenesis and metastasis. (**A**) Male. Tumor formation in the testis of a 1-day-old *Dnd1*-deficient male. 5× and 40× (**B**) Female. Neoplastic transformation in the ovary of *ter/ter* females at 5 and 10 days post partum. Top: 10× and 40×. Bottom: 2.5× and 40×. (**C**) Metastasis. Teratoma in adjunction to the vertebral column (white star). Tissue of ectodermal (squamous epithelium, black arrow), mesodermal (cartilage, gray arrow) and endodermal (glandular structures, white arrow) origin was identified. 2.5× and 10×.

Teratomas in WKY-*Dnd1^ter^* became clinically apparent during adolescence, however, the earliest formation of neoplastic tissue was found in newborn *ter/ter* testes at day 1 post partum, indicating an embryonic onset of tumorigenesis (Fig. 4A). No tumorous tissue has been exhibited by 1-day-old females to date (Fig. 4B), and the earliest tumorigenic tissue found in the ovary was at 5 days of age with more differentiated tumorigenic tissue observed in 10-day-old females (data not shown). From 897 *ter/ter* animals, metastatic spread was detected in the genital tract (seminal gland, epididymis or ductus deferens) and in the gut of 8 male and 1 female rat between 20 and 42 days of age. Furthermore, one female *ter/ter* rat had metastatic tumor growth in lung and liver at 128 days of age and a teratoma was identified at the base of the tail of one 40-day-old male rat. Histological analysis of this teratoma exhibited endodermal, mesodermal and ectodermal tissue in adjunction to the vertebrae (Fig. 4C).

### Testicular malformation in WKY-*Dnd1^ter^* males

**Figure 5:**
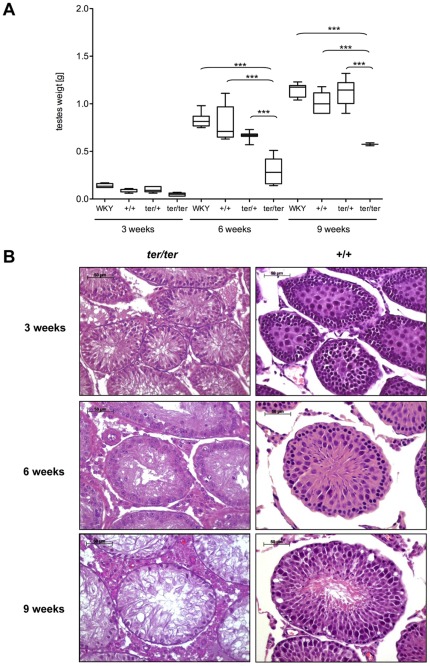
Non-tumorous testes. (**A**) Box plot analysis of the size of non-tumorous testes in WKY/Ztm and WKY-*Dnd1^ter^*/Ztm with wild type (+/+), heterozygous (*ter/+*) and homozygous (*ter/ter*) genotype at 3, 6 and 9 weeks of age. *ter/ter* testes are degenerated and significantly smaller than +/+ and *ter*/+ testes (***: p<0,001). (**B**) HE staining of wild type and homozygous *ter/ter* testes of WKY-*Dnd1^ter^*/Ztm rats from 3 to 9 weeks of age.

We defined the degenerated testis as lower in weight and size than the wild type testis (Fig. 1C, left). The mean weight of wild type testes increased from 0.14 g (SD±0.02) at 3 weeks to 0.83 g (SD±0.07) at 6 weeks and to 1.14 g (SD±0.07) at 9 weeks in WKY males, reflecting sexual maturation. In wild type WKY-*Dnd1^ter^* males the mean testes weight was 0.09 g (SD±0.02) at 3 weeks, 0.65 g (SD±0.05) at 6 weeks and 1.05 g (SD±0.14) at 9 weeks of age. There was no significant difference to heterozygous WKY-*Dnd1^ter^* males with 0.1 g (SD±0.03), 0.7 g (SD±0.04) and 1.12 g (SD±0.14) of testes weight at 3, 6 and 9 weeks of age. In *ter/ter* males the malformed, non-tumorous testes were reduced in size with 0.06 g (SD±0.01), 0.32 g (SD±0.13) and 0.81 g (SD±0.26) from 3 to 9 weeks of age (Fig. 5A). The ratio of unilateral degenerated testes decreased with age from 14.3% at 3 weeks to 9.5% at 6 weeks and to 7.1% at 9 weeks, while both testes were underdeveloped in 7% at 3 weeks, in 4.9% at 6 weeks and 2.4% at 9 weeks of age. The reduction of degenerated testes with age is directly correlated to the increase of teratoma formation in the *ter/ter* males.

**Figure 6:**
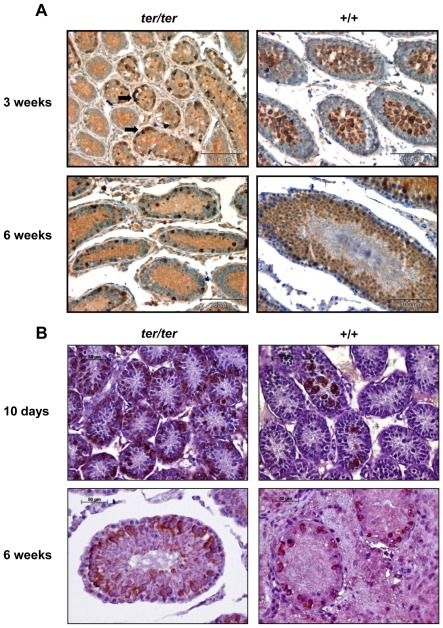
Immunohistochemical staining of testes. (**A**) anti c-kit staining and (**B**) anti DDX4/MVH staining of paraffin sections from homozygous *ter/ter* and wild type testes of WKY-*Dnd1^ter^*/Ztm rats at 10 days, 3 weeks and 6 weeks of age (black arrows: clusters of surviving c-kit positive germ cell). Abundance of germ cells detectable in +/+ testes, while few scattered clusters of positive cells remain in *ter/ter* testes.

Histological evaluation revealed normal testicular development and maturation in wild type and heterozygous WKY-*Dnd1^ter^* rats from 3 to 9 weeks. As expected, at 3 weeks of age wild type testes showed widely undifferentiated germ cell precursors in the seminiferous tubules, while at 9 weeks of age the complete differentiation process from spermatogonia to sperm could be observed. In *ter/ter* testes the failure of germ cell development was demonstrated by HE staining, which showed a reduced diameter of the tubuli seminiferi, a loss of germ cells and a lack of spermatogenesis at all ages (Fig. 5B). Furthermore, in some instances the degenerated *ter/ter* testes showed an onset of neoplastic transformation between the seminiferous tubules (Fig. 6B). No sperm could be recovered from *ter/ter* rats after the dissection of the epididymis (data not shown).

To confirm the loss of all sperm precursor cells, we performed immunohistological staining using an anti-c-kit antibody and anti-DDX4/MHV antibody as stem and germ cell markers. The various stages of germ cell development were detected by both antibodies in wild type testes from the ages of 10 days to 6 weeks (Fig. 6A, B). However, in the testes of both 3- and 6-week-old *ter/ter* rats only a few c-kit positive cells were detected in the tubuli seminiferi, and these remaining germ cells appeared to be aggregated to foci of neoplastic embryonic carcinoma cells (Fig. 6A). The same can be seen in the *ter/ter* testes stained against DDX4/MVH at 10 days and 6 weeks of age as the number of positive cells is sparse in comparison to the wild type tissue (Fig. 6B). The significant loss of germ cells on day 10 indicates a perinatal onset in the demise of germ cells as a response to the *Dnd1* mutation.

### Ovarian malformation in WKY-*Dnd1^ter^* females

**Figure 7:**
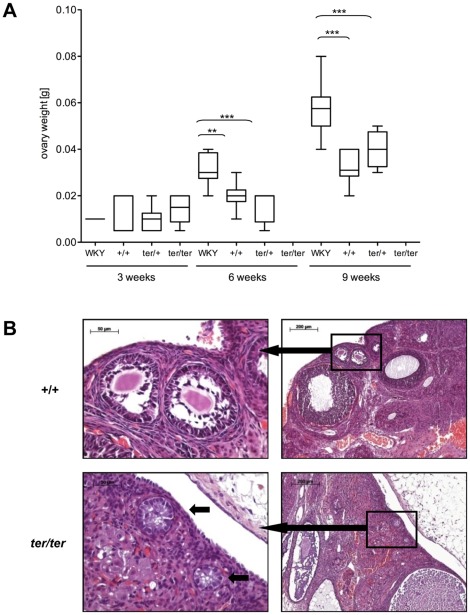
Non-tumorous ovaries. (**A**) Box plot analysis of non-tumorous ovaries from WKY/Ztm and WKY-*Dnd1^ter^*/Ztm with wild type (+/+), heterozygous (*ter*/+) and homozygous (*ter/ter*) genotype (**: p<0.01; ***: p<0.001). (**B**) HE staining of wild type (+/+, top panel) and mutant ovaries (*ter/ter*, bottom panel) of WKY-*Dnd1^ter^*/Ztm rats at 6 weeks of age. While different stages of follicle maturation (top panel, left) and primary follicles with a central oocyte (top panel, right) were evident in the wild type ovary, few follicle-like structures without germ cells (bottom panel, right, arrows) remained in the *ter/ter* ovary.

At 3 weeks of age, we found no significant differences in ovary size between females from the WKY or WKY-*Dnd1^ter^* strain (0.005–0.01 g). Three weeks later the ovaries grew to 0.032 g (SD±0.009) in WKY while the ovaries remained significantly smaller in WKY-*Dnd1^ter^* wild type (+/+) with 0.02 g (SD±0.006) and 0.015 g (SD±0.007) in heterozygous (*ter/+*) females. At 9 weeks of age the ovaries from WKY/Ztm females grew to 0.058 g (SD±0.011), in WKY-*Dnd1^ter^*/Ztm wild type (+/+) to 0.034 g (SD±0.007), and to 0.04 g (SD±0.007) in heterozygous (*ter/+*) females confirming sexual maturation (Fig. 7A). All *ter/ter* females had developed tumors at 6 and 9 weeks of age (Fig. 3C). Histological examination of early OGCTs in female *ter/ter* rats revealed no oocytes in the remaining non-neoplastic primary follicles, thereby indicating the loss of germ cells in females (Fig. 7B).

### *Dnd1* expression

**Figure 8:**
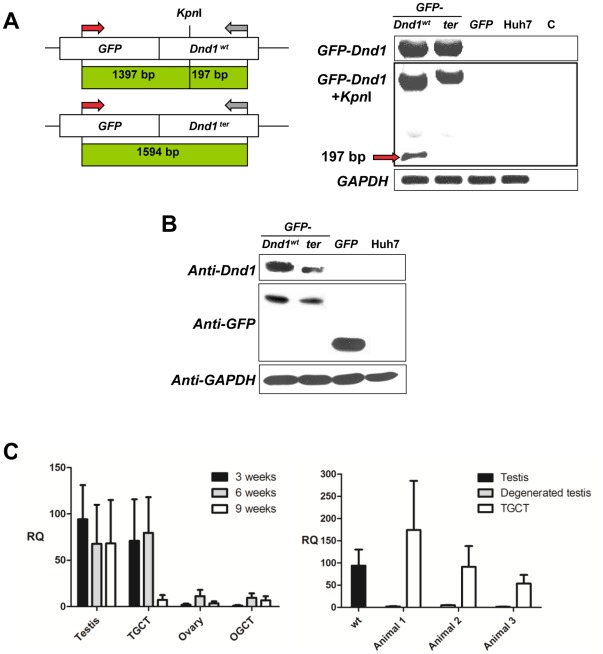
Dnd1 expression. (**A**) Huh7 cells were transfected with a cloned GFP-*Dnd1* construct and the cDNA was amplified by PCR. This was followed by *KpnI* restriction digest, to differentiate between the correct wild type and *ter Dnd1* cDNA. (**B**) The GFP-Dnd1 fusion proteins generated *in vitro* were detectable with an anti GFP and an anti Dnd1 antibody. Both the 66 kDa wild type and the 60 kDa ter GFP-Dnd1 fusion protein could be identified. (**C**) qRT-PCR. Left: *Dnd1* was transcribed in the gonads of animals at 3, 6 and 9 weeks of age. Higher levels were evident in testes compared to ovaries at all ages. Variable amounts were found in teratomas, ranging from high in TGCTs at 3 and 6 weeks, to low in TGCTs at 9 weeks and OGCTs at all ages. Right: *Dnd1* expression of three *ter/ter* animals with unilateral TGCT was measured in both the teratoma and the contralateral degenerated testis. The amount of *Dnd1* cDNA found in the teratomas was in the same range as wild type testes, while comparatively low levels were exhibited in the degenerated testis.

Cloning and expression of a Dnd1^ter^-GFP fusion protein (expected size: 60 kDa) resulted in a slightly truncated transcript compared to the 66 kDa Dnd1^wt^-GFP fusion protein. This was detectable by Western Blot analysis with both an anti-Dnd1 and an anti-GFP antibody (Fig. 8A, B).

*Dnd1* transcripts were identified by qRT-PCR in wild type and *ter* gonads at 3, 6 and 9 weeks of age. The number of *Dnd1* transcripts was affected by gender, and was higher in the cDNAs pooled from testes rather than ovaries. Transcription rate varied in teratomas, being low in the ovary at 3 to 9 weeks and the testis at 9 weeks and high in TGCTs at 3 and 6 weeks (Fig. 8C, left).

Furthermore, the *Dnd1* expression of three *ter/ter* animals with unilateral TGCT was measured in both the teratoma and the contralateral degenerated testis. The amount of *Dnd1* cDNA found in the teratomas was in the same range as wild type testes, while comparatively low levels were exhibited in the degenerated testis (Fig. 8C, right).

## Discussion

### Teratocarcinogenesis in the rat: a novel animal model for GCTs

Teratomas are rare tumors in rats and only a few cases of spontaneous teratocarcinogenesis have been reported in testis, adrenal gland, kidney, central nervous system or abdomen [43], [44], [45], [46]. Hereditary teratomas have also been found in testes and ovaries of the Tera strain that was developed from the Csk: Wistar-Imamichi rat [47]; however, the underlying mutation was never identified.

For unknown reasons the incidence of male GCTs and infertility are on the rise in humans, making it increasingly important to elucidate the mechanisms triggering these diseases. We detected a congenital, recessive point mutation referred to as *ter* in the rat *Dnd1* gene on chromosome 18p11 that induces teratocarcinogenesis and infertility in all homozygous animals of both genders. The mutant rat strain was denominated WKY-*Dnd1^ter^*/Ztm and it is a valuable new animal model for research on germ cell development, TDS, TGCTs and OGCTs.

The 129/Sv-*Ter* mouse is most likely a model for prepubertal type I TGCTs, whereas there are no animal models available for type II TGCTs [17], [48]. It remains to be established whether the teratomas of the *ter* rat show a closer resemblance to type I or type II GCT. However, in analogy to the mouse, and because the majority of rat teratomas occurred before or during puberty, it seems more plausible that the *ter* rat is a model for type I GCTs.

Neoplastic tissue was found as early as 1 day post partum in male neonates, indicating that a neoplastic transformation of germ cells takes place during embryogenesis. This correlates with the previously found results that primordial germ cells from embryos carrying the *ter* mutation are more easily transformed into pluripotent cells in culture than their wild type counterparts [36].

### Species and gender differences in *Dnd1*-related teratocarcinogenesis

In the WKY-*Dnd1^ter^*/Ztm rat, we identified a point mutation at position 1975 of the *Dnd1* gene that substituted G for A and introduced a premature stop codon causing a truncation of the Dnd1 protein, presumably at the c-terminus. A similar point mutation has been found in the mouse *Dnd1* gene with a C to T exchange in exon 2 on chromosome 18 that also generates a premature stop codon thought to result in a c-terminal truncation of the Dnd1 protein (Noguchi and Noguchi, 1985; Asada et al, 1996; Youngren et al, 2005). A rescue of Dnd1 in mutant *Ter* mice showed that the loss of functional *Dnd1* is responsible for the phenotype observed in the mouse. Dominant negative effects were excluded in the mouse, as tumors and germ cell deficient testes no longer expressed regular or truncated Dnd1 [25]. The close similarities found between *Dnd1* mutant mouse and rat in the mutation and the phenotype make it probable that a functional inactivation of *Dnd1* causes germ cell loss and teratomas in the *ter* rat.

Despite the similarities in the rodent *Dnd1* mutations, differences between the species mouse and rat were apparent in tumor incidence and severity of fertility disorders. In the mouse, *Dnd1* has been characterized as a modifier gene amplifying the incidence of spontaneous teratocarcinogenesis in heterozygous and homozygous *Ter* males. Other modifier genes (*Kitl* ^Sl-J^, *Trp53^nul^*, *A^y^*, M19, M19-A2, M19-C2) interact with mouse *Dnd1* and induce a 2–3 fold increase of TGCT incidence in heterozygous *Ter/*+ males [49]. Unlike the co-dominant *Ter* mutation in the mouse, the recessive *ter* mutation in rat *Dnd1* induced gonadal teratoma formation in 100% of the homozygous animals, while heterozygous and wild type animals completely lacked teratocarcinogenesis. Therefore, we postulate that *Dnd1* is an essential factor involved in teratocarcinogenesis and acts as a tumor suppressor gene in germ cells of the WKY/Ztm rat strain.

Furthermore, the malignant transformation of rat germ cells initiates not only testicular, but also ovarian teratocarcinogenesis. OGCTs developed in all homozygous *Dnd1*-deficient female rats, whereas no tumor development has been observed in female mice. In mice, male *Ter/Ter* germ cells fail to enter mitotic arrest in G0 [32]. The contrasting situation in the rat, with teratocarcinogenesis in both sexes, expands the role previously conferred to *Dnd1*, as the inability of germ cells to enter mitotic arrest in males does not offer an explanation for teratocarcinogenesis in females. The high level of the Dnd1 target gene *p27* found in mitotically arresting germ cells between d13.5 pc and 15.5 pc is not exhibited by male mutant mice or female germ cells between d12.5–14.5 pc [50]. Should the rat mirror the situation in the mouse, it would signify that either *Dnd1* target genes differ between females and males or that the downregulation of some cell cycle inhibitors might be a secondary effect and not the primary cause of tumor development. Therefore, the WKY-*Dnd1^ter^*/Ztm rat presents an exciting new model enabling future research on the various interactions of *Dnd1.*

Cook and colleagues demonstrated that the testicular environment is a crucial factor in GCT development of mice, by showing that female *Dnd1^Ter/Ter^* germ cells neoplastically transform in the testes [34]. The somatic cells of the testes secrete paracrine factors such as SCF, retinoic acid, FGF2, LIF, EGF and GDFN as well as androgenic hormones, and these might play a role in providing a tumor-promoting environment in the mouse [51]. The gender in the WKY-*Dnd1^ter^*/Ztm rats had no effect on tumor incidence, which was 100%, and only modified the tumor progression in homozygous animals. Surprisingly, on average tumors became clinically apparent earlier in females (35 d) where both ovaries were affected, while tumors developed slower in males (45 d) with about half of the tumors being unilateral. This accelerated tumor progression compensated the slightly delayed onset of teratoma formation seen in females compared to males. This could imply that, in the rat, the surroundings provided by the ovary might contain tumor-promoting factors or, alternatively, the testis might include inhibitory factors.

### *Dnd1* ablation causes germ cell loss and infertility

In response to the repression or ablation of *dnd* in *Xenopus laevis* and zebrafish embryos, PGCs failed to migrate into the developing gonads [26], [27]. A recent publication showed that the c-terminus of the zebrafish Dnd protein possesses an ATPase domain, which is essential for PGC formation and survival [52]. The presumed c-terminal truncation of Dnd1 in the mouse and rat was also linked to the ontogenetic loss of germ cells; however, it remains to be established which parts of the c-terminus are crucial for a functioning Dnd1 protein in rodents and whether an ATPase domain is involved. Immunohistochemical stainings show that the loss of PGCs starts in the embryo of *Dnd1* deficient mice of both genders [22]. Deficit of the PGCs caused abnormally small testes and male sterility in homozygous 129/Sv-*Ter* mice, while in the homozygous females a few germ cells survived and even matured to oocytes in ovaries of reduced size [21].

In contrast to the mouse, gametogenesis was absent in both degenerated testes and in non-neoplastic ovarian follicles of *ter/ter* rats, and this may result from the complete ontogenic loss of physiological germ cells. Differences in germ cell numbers were visible as early as day 10 post partum.

The WKY-*Dnd1^ter^*/Ztm rat strain exhibited infertility, small degenerated testis and TGCTs in males. The older the male *ter/ter* rats were, the lower the rate of degenerated testes and the higher the rate of bilateral tumors. Hence, it is highly likely that the infertile and degenerated testes exhibited by *ter/ter* males are the predecessors of cancer. This is substantiated by the fact that macroscopically degenerated testes exhibited neoplastic tissue between the tubuli seminiferi. The c-kit and DDX4/MVH positive cells remaining in the degenerated *ter/ter* testes are in all likelihood the pluripotent ECCs causing teratocarcinogenesis.

Every one of the *ter* females was infertile and all but one developed bilateral OGCTs without ever exhibiting any cases of degenerated ovaries, and this might be due to the faster teratoma growth in females compared to males.

### *Dnd1* expression

In the mouse *Dnd1* transcripts have been identified in heart, testis, and at a lower rate in TGCTs [25]. Rat *Dnd1* expression was highest in the gonads; however, it was not augmented in the rat heart compared to other organs, such as lung or spleen (data not shown).

Loss of germ cells and absence of gametogenesis in the degenerated testis is likely to be responsible for the low *Dnd1* expression exhibited by the three-week-old non-tumorous *ter/ter* testes. The amount of *Dnd1* present in GCTs was strongly variable. Teratomas are heterogeneous tumors, with none being like the other and often no longer resemble the organ from which they originated. Therefore, it is feasible that the distribution of *Dnd1* in a teratoma depends on the tissue predominant in the respective sample. Another possibility is that the *Dnd1* levels depend on, and as such would be indicative of, the number of undifferentiated ECCs present in the teratoma.

### Conclusion

While the mouse *Dnd1* was characterized as a modifier gene that modulates teratoma formation in males, our data suggests that rat *Dnd1* acts as a tumor suppressor gene in both genders of the WKY/Ztm strain. The WKY-*Dnd1^ter^*/Ztm rat provides a promising new model to study *Dnd1*-dependent GCT development in gonads of both genders and investigate the molecular mechanisms of GCT development in testes and ovaries. This model offers the opportunity to establish new therapeutic and diagnostic approaches for GCTs in men and women.
